# Attention Deficits Influence the Development of Motor Abnormalities in High Functioning Autism

**DOI:** 10.1007/s10578-020-01088-0

**Published:** 2020-11-04

**Authors:** Mariabernarda Pitzianti, Sabrina Fagioli, Marco Pontis, Augusto Pasini

**Affiliations:** 1 Unit of Child Neurology and Psychiatry, Department of Systems Medicine, “Tor Vergata” University, Via Montpellier 1, 00133 Rome, Italy; 2 Child Neuropsychiatry, USL Umbria-2, Viale VIII Marzo, 05100 Terni, Italy; 3 Department of Education, University of “Roma Tre”, Via del Castro Pretorio 20, 00185 Rome, Italy; 4 Neuroimaging Laboratory, IRCCS Santa Lucia Foundation, Via Ardeatina 306, 00179 Rome, Italy; 5 Comprehensive Rehabilitation Center Ctr Asl 8, Cagliari, Italy

**Keywords:** Autism, HFA, Attention and NSS, Selective attention, Attention deficits

## Abstract

Early attentional dysfunction is one of the most consistent findings in autism spectrum disorder (ASD), including the high functioning autism (HFA). There are no studies that assess how the atypical attentional processes affect the motor functioning in HFA. In this study, we evaluated attentional and motor functioning in a sample of 15 drug-naive patients with HFA and 15 healthy children (HC), and possible link between attentional dysfunction and motor impairment in HFA. Compared to HC, HFA group was seriously impaired in a considerable number of attentional processes and showed a greater number of motor abnormalities. Significant correlations between attention deficits and motor abnormalities were observed in HFA group. These preliminary findings suggest that deficit of attentional processes can be implied in motor abnormalities in HFA.

**Electronic supplementary material:**

The online version of this article (10.1007/s10578-020-01088-0) contains supplementary material, which is available to authorized users.

## Introduction

### Attentional Functioning in Autism Spectrum Disorder

Autism Spectrum Disorder (ASD) is a severe neurodevelopmental disorder diagnosed on the basis of persistent deficits in social communication and social interaction in different contexts and patterns of behavior or interests markedly restricted and repetitive [1]. As the symptoms of disorder fall on a continuum of severity, ASD is often divided into two forms: the first one with mental retardation and the second one with cognitive functioning average or above average, called high functioning autism or HFA [1]. In addition to the core symptoms of the disorder, attentional dysfunction is one of the most consistently reported deficits in patients with ASD, including subjects with HFA. Indeed, attentional abnormalities have been associated with the disorder since its first description [2]. Several studies show that patients with ASD exhibit early and pervasive abnormalities of attention [3–6]. Atypical attentional function has been shown in infants at risk for ASD and may be one of the earliest characteristics that distinguish infants who would later receive an ASD diagnosis [3, 4]. Most of the studies on attentional functioning in ASD are based on the Posner and Peterson conceptualization of attention as composite system. This system consists of three specialized neurofunctional networks, which are responsible for a distinct set of attention processes: the alerting, orienting and executive control networks [7]. Based on this model, abnormal function of each attentional network has been demonstrated in patients with ASD. Researches on alertness and arousal in ASD have been inconsistent: patients with ASD exhibit intact tonic [8] and phasic [9] components of alerting, yet demonstrate atypical arousal [10] and reduced sensitivity to novel information [11]. According to the study conducted by Dawson and colleagues, patients with ASD have difficulties orienting to both social and non-social information within their environment [12]. According to studies conducted by other authors, individuals with ASD have difficulties disengaging [13] and shifting visual attention [14] and show atypical activation of the orienting network [15]. Further studies confirm that patients with ASD show significant impairments in disengaging visual attention [3, 16]. Finally, studies on executive control abilities in ASD suggest intact inhibitory processing [17, 18], but impaired cognitive flexibility [19, 20]. Together, these findings indicate that individuals with ASD exhibit lifelong abnormalities in the adaptive allocation of visual attention.

### Motor Functioning in Autism Spectrum Disorder

Motor impairment has been widely reported in patients with ASD, including subjects with HFA. Several studies have found deficits in many aspects of motor function, including coordination, gait and motor preparation in children and in adults with ASD. Motor dysfunction may lead to great difficulty for subjects with ASD in negotiating their physical environment, fine motor control (e.g. writing and tying shoes) and social play (e.g. riding a bike, throwing a ball and participating in team sports) [21]. In a study of motor control, children with ASD showed impaired performance on a wide variety of measures of motor examination, as compared to a control group of same-age subjects; in that study patients with ASD showed greater difficulty with balance and gait, slower speed and more dysrhythmia with timed movements of hands and feet and greater overflow movements during performance of timed movements and stressed gait maneuvers [22]. According to previous studies, we found that children with Asperger syndrome (included in ASD) and HFA experience motor impairment and a variety of neurological soft signs (NSS) [23, 24]. Neurological Soft Signs (NSS) are subtle motor, sensory and integrative abnormalities that cannot be related to impairment of a specific brain region and result in considerable sociopsychological dysfunction [25]. Although NSS are commonly observed in children with typical development and reflect the immaturity of the central nervous system, their persistence into later childhood and adolescence suggests motor dysfunction and could be a “marker” of atypical neurodevelopment [26]. NSS are mainly represented by overflow movements (OM) and dysrhythmia. OM are defined as co-movements of body parts not specifically needed to efficiently complete a motor task [26]. There are a number of different forms of OM: associated movements, contralateral motor irradiation and mirror movements. Associated movements refer to involuntary movement in non-homologous muscles, either contralaterally or ipsilaterally [27]. Contralateral motor irradiation and mirror movements are involuntary movements that occur in the homologous muscles contralateral to voluntary movements [28]. Dysrhythmia is defined as an improper timing and/or rhythm of movement otherwise normal [29]. OM seem to be related to a delay or defect of maturation within the intra-cortical and inter-cortical systems that support automatic inhibition [30], while dysrhythmia appears to be caused by cerebellar dysfunction [31]. Dysrhythmia and slowness of timed movements of hands and feet seem to be the most prominent motor abnormalities in children with ASD, including subjects with Asperger syndrome and HFA [22–24, 32] and to reflect functional deficits of the frontostriatal system, cerebellum and basal ganglia [23, 24, 31, 32].

### What This Paper Adds

Starting from the conceptualization of attention devised by Posner and Petersen [7], Van Zomeren and Brouwer delineated a multidimensional model of attention, including tonic and phasic alertness, vigilance/sustained attention, selective attention, divided attention and strategy/flexibility [33]. While tonic alertness refers to a relatively stable level of attention which changes slowly according to diurnal physiological variations of the organism, phasic alertness is the ability to enhance the activation level following a stimulus of high priority. Selective attention is defined as the ability to focus attention in the face of distracting or competing stimuli. Divided attention requires a simultaneous response to multiple tasks or multiple task demands. The ability to sustain attention enables a subject to direct attention to one or more sources of information over a relatively long and unbroken period of time [33]. This multidimensional model also encompasses the distinction between aspects of selectivity and intensity made by Kahneman [34] and the concept of a supervisory attentional control devised by Shallice [35]. To our knowledge, studies on attentional functioning in ASD based on this multidimensional model are not available. Therefore, we have chosen to assess several components of attention, as suggested by the multicomponent model of Van Zomeren and Brouwer, in a drug-naive sample of children with HFA as well as in healthy peers. We have evaluated motor functioning by the Physical and Neurological Assessment of Subtle Signs (PANESS) [36] in patients with HFA and compared them to healthy subjects. The role of atypical attentional networks in the emergence of autism has been widely described (Keehn et al. [37] for a review) as well as the influence of attentional processes on motor functioning in normal school-age children [38–40]. To our knowledge there are no studies that investigated the co-occurrence of atypical attentional processes and NSS in clinical populations with HFA. The present study strives to fill the gap by analyzing the relationship between attentional functioning and motor impairment in subjects with HFA.

## Method

### Participants

The study included 30 participants divided into a clinical group and a control group: 15 patients with HFA (13 boys, 2 girl) and 15 healthy controls (12 boys, 3 girl) aged 7–16 years with an IQ ≥ 85. The subjects in the clinical group were consecutive referrals of the Unit of Child Neurology and Psychiatry of Tor Vergata University of Rome, Italy. In accordance with the DSM-5 criteria [1], the diagnosis of HFA was based on clinical assessment, observation of children and interview with parents, which were carried out by an experienced child psychiatrist. The Autism Diagnostic Inventory-Revised (ADI-R) [41] and the Autism Diagnostic Observation System (ADOS) [42] were used to make the diagnosis of HFA. The long version of the Conners’ Parents Rating Scale-Revised (CPRS-R) and the Conners’ Teachers Rating Scale-Revised (CTRS-R) [43] and the interview with the Schedule for Affective Disorders and Schizophrenia for School-Age Children–Present and Lifetime Version (K-SADS-PL) [44] were used to exclude the diagnosis of Attention Deficit Hyperactivity Disorder (ADHD) and other psychiatric comorbidities in all patients with HFA. The healthy children were recruited in schools and selected from a pool of subjects who participated voluntarily in the study. None of them had a history of neurological or psychiatric disease or learning disability. The long version of the Conners’ Parents Rating Scale-Revised (CPRS-R) and the Conners’ Teachers Rating Scale-Revised (CTRS-R) [43] and the interview with the Schedule for Affective Disorders and Schizophrenia for School-Age Children–Present and Lifetime Version (K-SADS-PL) [44] were used to exclude the diagnosis of ADHD and other psychiatric disorders in all healthy participants. All subjects included in the study had a normal IQ as measured with the Wechsler Intelligence Scale for Children-III (WISC-III) [45]. At the time of the study, no participants were taking any medication known to affect the central nervous system. Before the testing and in accordance with the Helsinki Declaration, every parent or legal guardian of the subjects included in the study undersigned a written informed consent.

### Evaluation of Attentional Functioning

All participants were tested with four computerized tasks measuring different aspects of attention. The tests of attention used were developed and validated for the assessment of attentional deficits in children and adults with cerebral lesions [46, 47]. Test procedures were presented on a computer screen. Instructions were given orally by an examiner who was not blinded to the group membership. Albeit the participants’ clinical status was not fully blinded to the examiner, the computerized test battery administration (i.e., Test of Attentional Performance (TAP; Zimmermann and Fimm [47])), that has typically high objectivity in terms of administration and scoring, minimized the effect of any putative bias in the examiner’s assessment.

Participants were instructed to perform the computerized tasks as quickly as possible maintaining a high level of accuracy. In order to familiarize the participants with the tasks, two brief sequences of about five practice trials preceded each test. Tests were performed only after participants had completed the practice trials without errors. All the participants were able to perform the actual task without additional training. There was no difference between HFA and healthy participants in the number of practice trials needed to understand the tasks.

Participants were assessed individually in a quiet room and the examiner was present during the entire assessment.

In the *alertness task*, reaction time is examined under two conditions. The first condition represents a simple reaction time measurement, in which a cross appears on the monitor at randomly varying intervals and to which the subject has to respond as quickly as possible by pressing a key, providing a measure of intrinsic alertness. In the second condition, reaction time is measured in response to a critical stimulus preceded by a cue stimulus presented as warning tone (“phasic arousal” or temporal orientation of attentional focus). Therefore, in the alertness task participants were asked to respond by pressing a button when a visual stimulus appeared on a computer screen. In the first 20 trials, the stimulus appeared on the screen without prior warning (tonic alertness task), while during the second 20 trials, a warning tone preceded the appearance of the stimulus (phasic alertness task). The time span between the warning tone and the appearance of the stimulus was random [48]. Measures of tonic and phasic alertness are calculated on the basis of the reaction time of the participant. In addition, the variability of reaction time and number of omission errors are measured.

The *incompatibility task* tests the interference tendency in terms of stimulus-reaction incompatibility. For this test, arrows that are directed to the left or the right are presented on the left or right side of a fixation point. Depending on the direction of the arrow, the tested person is requested to respond with the right or left hand irrespective of the side on which the arrow is presented. Therefore, in the incompatibility task, arrows pointing to the left or the right were presented briefly on the left or right side of a fixation point in the center of the computer screen. The participants were requested to press a response button as quickly as possible on the side indicated by the direction of the arrow, independent of the position of the arrow. If the position of the arrow and its orientation accorded (e.g. arrow on the left side of the fixation point pointing to the left side), the trial was classified as a compatible trial while trials in which presentation and orientation were not in accordance (e.g. arrow on the left side of the fixation point pointing to the right side) were classified as incompatible trials. The sequence of trials was random, with about half of the trials compatible and half incompatible [48]. Reaction time, variability of reaction time and number of commission errors are calculated, providing a measure of selective attention as the capacity to reject irrelevant information.

The *divided attention task* requires participants to process in parallel a visual and an auditory task presented by a computer. In the visual task, a series of matrices was presented in the center of the computer screen. Each matrix, consisting of a regular array of sixteen dots and crosses (4 × 4), was displayed for 2000 ms. The participant was asked to press the response button as quickly as possible whenever the crosses formed the corners of a square (visual target). In the acoustic task, the participant was requested to listen to a continuous sequence of alternating high and low sounds and to press the response button as quickly as possible when irregularities of the sequence occurred (acoustic target) [48]. Reaction time for correct responses, variability of reaction time and number of omission errors (lack of response to target stimuli) and number of commission errors (responses to non-target stimuli) are calculated as a measure of divided attention.

In the *sustained attention task*, a sequence of stimuli is presented on the monitor. The stimuli vary in a range of feature dimensions: color, shape, size and filling. A target stimulus occurs whenever it corresponds in one or the other of two predetermined stimulus dimensions with the preceding stimulus (e.g. the same shape but with different color, size and filling). Different levels of difficulty may be selected (e.g. reaction only to “shape” or reaction to “colour and shape”). In order to adapt the difficulty of the task to the performance level of the subjects, reaction only to “shape” was chosen [48]. Reaction time for correct responses, variability of reaction time, number of omission errors (lack of response to target stimuli) and number of commission errors (responses to non-target stimuli) are calculated as a measure of sustained attention.

### Assessment of Neurological Soft Signs

For the assessment of motor functioning, the *Physical and Neurological Assessment of Subtle Signs* (PANESS) [36] was applied. These evaluations were performed by a child neurologist who underwent training for the reliable application of the PANESS. The examiner was blind to the child’s diagnostic status at the time of assessment and during scoring. The PANESS has been found to have adequate test–retest reliability [49], inter-rater reliability, internal consistency [50] and sensitivity to age-related changes [26] in more current and diverse cohorts. The PANESS measures salient components of motor function, including lateral preference, gaits, balance, motor persistence, coordination, overflow, dysrhythmia and timed movements. Three primary outcome variables were obtained: (1) *total overflow movements* included the total number of abnormal movements for age observed during stressed gaits (i.e. walking on heels, toes or sides of feet), tandem gaits (walking in tandem forward and backward, touching heels to toes) and during timed movements; (2) *total dysrhythmia* included total number of times in which the children failed to maintain a steady rhythm throughout the task; (3) *total speed of timed activities* of hands/feet included three repetitive movements and three sequenced movements which were performed bilaterally: toe tapping, alternating heel-toe tapping, repetitive hand patting, hand pronation/supination, repetitive finger tapping and finger sequencing.

### Statistical Analysis

Statistical analyses were performed using IBM SPSS Statistics version 21. Group difference on the gender variable was assessed with the χ^2^ test. Independent t-tests were used to assess differences between HFA participants and HC in terms of age, IQ and presence of soft motor signs. Differences between the two groups on the attention functioning were also tested through independent t-tests comparisons. Specifically, we first computed the mean reaction times (RTs), the mean variability of reaction times and the mean number of errors for each subject for each of the four attention tasks: (a) Alertness (tonic vs phasic) (b) Selective attention (c) Divided attention (visual vs auditory) and (d) Sustained attention. Then, the performance scores of HFA and HC groups were compared using unpaired two-sample t-tests. A t statistic corrected for the non-homogeneity of variance was computed when Levene’s test for equality of variances reached significance.

Cohen’s *d* was used to calculate the effect size for differences between paired observations [51]. Following Cohen’s (1988) guidelines for interpreting effect sizes, small effects (*d* ≥ 0.20), medium effects (*d* ≥ 0.50) and large effects (*d* ≥ 0.8) were distinguished [52]. In order to account for the small sample size (n = 15), we also run non-parametric Mann–Whitney U test and find similar results to those discussed here. This non-parametric analysis is described in the Supplementary Material. The level of statistical significance used for all the analyses was defined as p < 0.05.

Finally, to determine the significance of the correlations between soft motor signs and variables of attentional functioning, correlational analyses and Fisher’s r to z transformation were performed.

## Results

### Demographic and Neurological Characteristics

Demographic and neurological characteristics of HFA and HC are illustrated in Table 1. In our study patients and healthy controls did not differ in terms of age, gender and IQ (Table 1, Panel A). Instead, we found significant differences between the HFA and control groups with regard to total overflow movements, total dysrhythmia and total speed of timed activities (Table 1, Panel B).

**Table 1:** Demographic and neurological characteristics of 15 participants with HFA and 15 healthy control subjects

A. Demographical variables					
Variable	HFA (n = 15)	Controls (n = 15)	*t*	*df*	*p*
	Mean (SD)	Mean (SD)			
Age	10.53 (2.1)	11.6 (2.77)	− 1.188	28	0.245
IQ	104.07 (12.38)	100.87 (8.93)	0.812	28	0.424

	N (%)	N (%)	χ^2^	*df*	*p*
Gender (male)	13 (87)	12 (80)	0.24	1	0.624

B. Motor assessment using PANESS					
Variable	HFA (n = 15)	Controls (n = 15)	*t*	*df*	*p*
	Mean (SD)	Mean (SD)			
Total overflow movements	3.53 (1.24)	1.73 (1.95)	3.019	23.84	**<** **0.01**
Total dysrhythmia	11.60 (1.55)	1.80 (1.74)	16.29	28	**<** **0.01**
Total speed of timed activities	202.27 (39.17)	157.07 (14.19)	4.202	28	**<** **0.01**

*d.f* degrees of freedom, *SD* standard deviation

Significant p values are indicated in bold

### Attentional Functioning

Performance scores of HFA and HC on the four attention tasks are reported in Table 2.

**Table 2:** Performance on attention tasks of 15 participants with HFA and 15 healthy control subjects

Variable	HFA (n = 15)	Controls (n = 15)	*t*	*df*	*p*	*d*
	Mean (SD)	Mean (SD)				
A. Alertness						
Tonic alertness						
Mean RTs	349.07 (102.08)	312.13 (101.48)	0.994	28	0.329	0.36
Variability of RTs in msec	94.47 (59.38)	51.20 (40.70)	2.328	28	**0.027**	0.85
Number of omission errors	0.20 (0.41)	0.07 (0.26)	1.058	23.46	0.301	0.38
Phasic alertness						
Mean RTs	314.00 (101.93)	281.47 (83.96)	0.954	28	0.348	0.35
Variability of RTs in msec	72.53 (69.42)	39.13 (19.12)	1.797	28	0.083	0.65
Number of omission errors	0.07 (0.26)	0.07 (0.26)	< 0.001	28	1.00	0.00
B. Selective attention						
Mean RTs	571.53 (97.47)	568.60 (124.99)	0.49	28	0.962	0.02
Variability of RTs in msec	139.60 (44.01)	99.27 (30.86)	2.906	28	**0.007**	1.06
Number of omission errors	2.53 (4.63)	0.27 (0.59)	1.882	14.461	0.080	0.68
C. Divided attention						
Auditory task						
Mean RTs	788.20 (303.19)	652.87 (83.25)	1.667	16.099	0.115	0.61
Variability of RTs in msec	290.53 (190.61)	141.07 (56.96)	2.910	16.480	**0.010**	1.06
Number of omission errors	1.87 (1.24)	0.53 (0.52)	3.829	18.672	**0.001**	1.41
Visual task						
Mean RTs	982.20 (169.66)	902.00 (124.75)	1.475	28	0.151	0.54
Variability of RTs in msec	391.67 (146.91)	243.93 (111.95)	3.098	28	**0.004**	1.13
Number of omission errors	3.87 (2.72)	1.53 (1.35)	2.972	20.543	**0.007**	1.09
Total number of errors	31.93 (43.12)	3.27 (4.20)	2.563	14.266	**0.022**	0.93
D. Sustained attention						
Mean RTs	678.93 (218.81)	666.40 (133.64)	0.189	28	0.851	0.07
Variability of RTs in msec	216.93 (106.65)	167.87 (79.05)	1.431	28	0.163	0.52
Number of omission errors	20.67 (13.13)	3.80 (4.19)	4.739	16.829	**<** **0.001**	1.73
Number of commission errors	52.13 (41.64)	5.40 (6.03)	4.302	14.587	**0.001**	1.57

*d.f* degrees of freedom, *SD* standard deviation, *RT* reaction times

Significant p values are indicated in bold

#### Alertness

Comparison between the HFA and control groups using unpaired two-sample t-tests revealed no significant differences with regard to reaction time and number of omission errors in the tonic alertness task; however, we found that variability of reaction time was greater in the HFA group than healthy controls. The performance on the phasic alertness task did not differ significantly between the two groups (Table 2, Panel A).

#### Selective Attention

There were no significant differences between the HFA and control groups with regard to reaction times and the number of omission errors in the incompatibility task, but we found significant difference in the variability of reaction time between the two groups within the same task. Again, we found that HFA showed greater variability in RTs than healthy participants (Table 2, Panel B).

#### Divided Attention

In the auditory task we found significant difference between the HFA and control groups with regard to variability of reaction time and number of omission errors, but not with regard to reaction time. Specifically, HFA participants showed greater variability of reaction times and committed a higher number of missing responses than HC participants. Similarly, in the visual task, we found that children with HFA showed greater variability of reaction times and more omission errors than HC. Finally, we found that, in the overall performance, HFA patients were less accurate than HC (Table 2, Panel C).

#### Sustained Attention

In the sustained attention task, the HFA group did not differ from the control group in reaction time and variability of reaction time, but the patients with HFA showed a greater number of both omission and commission errors when compared to the healthy controls (Table 2, Panel D).

### Correlations Between Attentional and Motor Performances Within the Clinical Group

Bivariate correlations between soft motor signs and performance on the four attention tasks are reported in Table 3.

**Table 3:** Bivariate correlation between soft motor sign and attentional functioning in 15 patients with HFA and 15 healthy control subjects

Variables	HFA (n = 15)		
	Total overflow movements	Total dysrhythmia	Total speed of timed activities
A. Alertness			
Tonic alertness			
Mean RTs	0.296 (0.284)*	0.056 (0.843)	0.381 (0.161)
Variability of RTs in msec	0.089 (0.752)	0.062 (0.827)	− 0.245 (0.379)
Number of omission errors	**0.583 (0.023)**	**0.544 (0.036)**	− 0.193 (0.490)
Phasic Alertness			
Mean RTs	0.379 (0.163)	0.341 (0.213)	0.166 (0.553)
Variability of RTs in msec	0.133 (0.636)	0.258 (0.354)	0.020 (0.944)
Number of omission errors	0.161 (0.566)	0.291 (0.293)	− 0.372 (0.172)
B. Selective attention			
Mean RTs	0.167 (0.551)	− 0.073 (0.797)	0.324 (0.239)
Variability of RTs in msec	0.082 (0.772)	− 0.185 (0.510)	**0.555 (0.032)**
Number of omission errors	− 0.078 (0.783)	0.283 (0.307)	0.127 (0.651)
C. Divided attention			
Auditory task			
Mean RTs	− 0.035 (0.900)	0.146 (0.605)	0.052 (0.854)
Variability of RTs in msec	0.197 (0.481)	0.280 (0.312)	0.048 (0.864)
Number of omission errors	0.290 (0.295)	0.159 (0.572)	0.179 (0.524)
Visual task			
Mean RTs	− 0.428 (0.112)	− 0.453 (0.090)	0.113 (0.689)
Variability of RTs in msec	**0.534 (0.040)**	0.358 (0.190)	0.293 (0.289)
Number of omission errors	− 0.134 (0.634)	− 0.262 (0.345)	0.159 (0.572)
Total number of errors	**0.583 (0.023)**	**0.605 (0.017)**	0.067 (0.814)
D. Sustained attention			
Mean RTs	0.195 (0.485)	0.235 (0.399)	0.277 (0.317)
Variability of RTs in msec	0.353 (0.197)	**0.644 (0.010)**	− 0.102 (0.717)
Number of omission errors	**0.613 (0.015)**	0.160 (0.569)	0.267 (0.336)
Number of commission errors	0.334 (0.223)	0.154 (0.584)	− 0.154 (0.584)

*Spearman Rho sr (p value); Significant p values are indicated in bold

Regarding alertness, the analysis showed a significant positive correlation between the total number of omission errors in the tonic alertness task and greater measure of overflow movements and dysrhythmia (Table 3, Panel A).

Regarding selective attention, a significant correlation was found between the variability of reaction time and total speed of timed activities (Table 3, Panel B).

Regarding divided attention, the analysis revealed a significant positive correlation between the variability of reaction time and overflow movements in the visual task. Moreover, the total number of errors in the overall performance (i.e., considering the visual and auditory tasks together) correlated positively with the total overflow movements and dysrhythmia (Table 3, Panel C).

Regarding sustained attention, we found that variability of reaction times was associated with higher measure of dysrhythmia in HFA children. Furthermore, the analysis revealed that low accuracy (i.e., measured in terms of omission) was associated with higher overflow movements (Table 3, Panel D).

No other correlations reached statistical significance.

## Discussion

The neuropsychological theories of autism suggest contributions of attentional deficits to the development of social communication problems [53, 54]. Moreover, in ASD procedural learning mechanisms, important for acquisition of motor skills, may also contribute to impaired development of communicative and social skills [55–57]. Therefore, the motor signs exhibited by individuals with ASD may serve as markers for deficits in parallel systems important for communication and socialization [55–57]. In this framework the careful consideration of attentional and motor impairments may provide valuable information about the neurobiological basis of ASD. Furthermore, the correlation analysis between attentional and motor functioning may help to clarify how the atypical attentional processes may be related to the motor signs in HFA. Therefore, the present study has had two main aims: the first one was to evaluate extensively attentional functioning and motor signs in a drug-naive sample of children with HFA compared to healthy children; the second one was to elucidate a potential relationship between attentional and motor performances in HFA. To the best of our knowledge, this is the first study in which attentional functioning was assessed according to the multidimensional model devised by Van Zomeren and Brouwer [33]. All participants were assessed with a computerized tests battery, which measures different aspects of attention, such as tonic and phasic alertness, selective attention, divided attention and sustained attention. Finally, a correlation analysis was conducted to evaluate the relationship between attention deficits and the salient components of motor function—as measured by PANESS—in HFA.

### Attentional Dysfunction in HFA

The present study reveals that, compared to healthy sex- and age-matched children, patients with HFA were impaired in a considerable number of attentional processes, including alertness, selective attention, divided attention and sustained attention. While selective attention and divided attention are considered to be aspects of selectivity, alertness and sustained attention are expressions of intensity of attention [33]. Therefore, attentional dysfunction observed in our patients involves both aspects of selectivity and intensity of attention. Compared to healthy subjects, children with HFA displayed an enhanced variability of reaction time in tasks of tonic alertness, in those of selective attention, as well as in the auditory and visual tasks of divided attention. The variability of reaction time is considered a measure of the attentional fluctuations in the subject’s efficiency of processing during the course of a continuous task [33]. The present study showed that, in comparison to healthy peers, children with HFA displayed a significant impairment of accuracy in divided and sustained attention tasks, but not in those of alertness and selective attention. Indeed, a greater number of omission errors in divided attention task and a greater number of omission and commission errors in the sustained attention task were observed in the HFA group compared to the control group. While omission errors (lack of response to target stimuli) are considered as a measure of inattention, commission errors (responses to non-target stimuli) are a measure of impulsivity [58, 59]. The goal of this study is to demonstrate the presence of attentional dysfunction in individuals with HFA using for the first time the multidimensional model devised by Van Zomeren and Brouwer. The body of this research shows that ASD is characterized by attentional dysfunction of the alerting, orienting and executive control networks. Moreover, ASD may be characterized by dysmodulation of arousal (subjects with hyperarousal and subjects with hypoarousal) and impaired novelty processing, slowed attentional disengagement and shifting and poorer performance on complex executive control tasks [37]. Attentional processes are mediated by cerebral networks, including several cortical and subcortical brain regions. Alertness functions are supported by the locus coeruleus-norepinephrine system (LC-NE), which is the core arousal center. Through its projections to the thalamic nuclei and cerebral cortex, the LC-NE supports appropriate levels of alertness in order to maintain efficient information processing [60]. The anterior cingulate cortex and the right dorsolateral prefrontal frontal cortex maintains tonic alertness by modulating activity in the locus coeruleus via the reticular nucleus of the thalamus [61]. The alerting network is mediated by a right-lateralized ventral frontoparietal network, which is responsible for achieving and maintaining appropriate levels of alertness [62]. Selective attention is related to anterior cingulate gyrus, inferior frontal cortex (left hemisphere) and frontal-thalamic connections to the reticular nucleus of thalamus [63]. Divided attention is served by frontoparietal network [64, 65] and anterior cingulate gyrus [66]. Sustained attention is mediated by reticular and intralaminar thalamic nuclei and anterior cingulate gyrus [63].

According to the results of the present study, it may be hypothesized that a dysfunction of these cerebral networks is involved in early and lifelong abnormalities in efficiently modulating attentional processes in patients with ASD, including subjects with HFA.

### Motor Impairment in HFA

The present study reveals that, in comparison to healthy sex- and age-matched children, patients with HFA performed worse on the Physical and Neurological Assessment of Subtle Signs (PANESS) [36], used to assess motor functioning. Indeed, the HFA group showed multiple motor abnormalities as compared to the control group. These abnormalities included a greater number of OM, a greater dysrhythmia and a greater motor slowness. These findings are consistent with results of previous investigations, that emphasize the presence of motor dysfunction in children with ASD. According to these studies, patients with HFA exhibit difficulty with motor preparation and execution, increased dysrhythmia and motor slowness of time activities, when compared to healthy children [22–24, 32]. These motor impairments could reflect functional deficits in the fronto-striatal circuits and cerebellum and dysfunction of basal ganglia [67, 68]. Functional magnetic resonance studies found that patients with ASD have greater variety in their functional maps and less distinct regional activation patterns than the healthy controls. Therefore, motor pathways are not properly organized in subjects with ASD, leading to difficulties in generating appropriate motor responses [69]. In particular some functional imaging studies showed an atypical activation on the premotor cortex [70] and the cerebellum [71–73], during motor execution and decreased connectivity of the motor execution network [73]. Cardinale and colleagues reported atypical rightward lateralization of multiple functional brain networks in subjects with ASD, including language, motor and visuospatial circuits [74]. This result was confirmed recently: indeed, in a study conducted by Floris and collaborators in 2016, children with ASD showed rightward lateralization in mean motor circuit connectivity compared to typically developing children and this was associated with poorer performance on all three PANESS measures [75]. Structural magnetic resonance studies revealed an increased brain volume in younger, but not older, children with ASD [76–78]. The increased volume has been principally attributed to larger white matter volumes, particularly in outer radiate regions [79]. Mostofsky and colleagues found a robust positive correlation between total PANESS score and left hemisphere primary motor and pre-motor white matter volume. According to the same authors, the correlation between motor performance and left motor cortex white matter volume distinguishes children with ASD from typically developing children and from those with ADHD. Therefore, the authors believed that an increased radiate white matter volume within the primary motor cortex may be a predictor of motor impairment in children with ASD [80]. It may be hypothesized that overgrowth of localized cortical connections and undergrowth of more distant connections between cerebral cortical regions and subcortical structures [79, 81] result in impaired complex information processing and weak central coherence [82, 83] and also contribute to impaired motor sequence learning necessary for development of complex motor skills and social/communicative gestures [55–57, 84].

### Neurological Soft Signs and Attentional Dysfunction in HFA

The influence of attentional processes on motor performance has already been studied in non-clinical populations [38–40]. Waber and colleagues analyzed the role of attentional processes in OM in normal school-age children. According to these authors, children who produced high levels of OM were more responsive to task-irrelevant cues (maintained low levels of attention), whereas those who produced low levels of OM were more responsive to task-relevant cues (maintained high levels of attention) [38]. The relationship between reduced attention and increased OM was strengthened by subsequent findings of a reduction but not elimination of OM when participants were asked to inhibit it [39]. Moreover, a study by Lazarus and Todor showed that children of all age groups reduced the magnitude of OM when receiving sensory feedback during the task [40]. The goal of the present study was to analyse the relationship between attentional processes and motor functioning in the clinical population with HFA. Using correlation analysis, we found significant correlations between disturbances of attentional functioning and motor abnormalities in children with HFA. Based on our results, deficit of alertness (in terms of increased number of omission errors) correlates significantly with increased OM and greater dysrhythmia. Dysfunction of selective attention (in terms of variability of reaction time) correlates significantly with greater motor slowness of time activities. Disturbance of divided attention (in terms of variability of reaction time in visual tasks and increased number of commission errors in both auditory and visual task) and impairment of sustained attention (in terms of increased number of omission errors) correlates with increased OM. Furthermore, disturbance of divided attention (in terms of increased number of errors in both auditory and visual task) and impairment of sustained attention (in terms of increased variability of reaction time) correlate significantly with greater dysrhythmia.

Since the reaction time, the variability of reaction time and the number of omission errors are considered a measure of inattention, our findings suggest that a link is present between attentional and motor functioning in children with HFA. Our results support the hypothesis that impairment of the core components of attention control—i.e., alertness, orienting, divided and sustained attention—plays a role in the pathophysiology of the NSS in clinical population with HFA. Indeed, several studies on motor learning showed that motor abnormalities in autism may be secondary to a deficit in executive functions, such as planning [32, 85] and learning skills [55], rather than general motor abilities. For instance, it has been showed that individual with autism have difficulty in learning the sequence of movements necessary to perform skilled motor tasks [55]. In addition, they exhibit deficits in the preparation of an action, whereas they have intact ability to execute action [32]. Consistent with these studies, our results may suggest that atypical motor functioning can be secondary to executive dysfunction possibly involving difficulty in disengaging the orienting of attention from a target object [86] or difficulty in selecting the proper movement for the action preparation. Intriguingly, as we revealed an association between atypical motor functioning and attention control through a correlational analysis, it is conceivable to hypothesize that attention deficits are indeed secondary to motor impairment. Several studies showed that individuals with autism place unusual reliance on proprioception when learning a novel movement pattern [87, 88], and exhibit difficulty to integrate visual spatial and temporal characteristics of a movement to guide and adjust motor tasks, like catching or throwing a ball [89]. Motor symptoms occur early in ontogeny and may precede the development of the core features of the disorder [90]. In this vein, atypical internal model of the action that place strong reliance on proprioception may lead to attentional deficits involving erroneous prediction of the sensory consequence of self-generated action, thus impairing skill development [87, 88]. Deeper examination of the relationships between impairment of the core components of attention control and motor abnormalities in autism may contribute to further define motor impairment specificity in children with HFA.

## Conclusions

So far, attentional functioning studies in ASD were addressed to understanding the role of atypical attentional processes in the emergence of socio-communicative impairment typical of the disorder. To the best of our knowledge, this is the first study in which the correlation between attentional dysfunction and motor impairment was analyzed in a clinical population with HFA. Our findings suggest that impairment of the core components of attention control is related to abnormalities of salient characteristics of motor function in individuals with HFA. The first strength of our study is the inclusion of a well-defined group of drug-naive children with HFA, who were carefully screened for other comorbid psychiatric conditions. The second strength of our study is the inclusion of subjects with normal IQ. Indeed, lower IQ appears to be related to increased NSS in children with HFA.

The interpretation of our findings is limited by the small sample size, and the study should be qualified as exploratory in nature. Further studies on a greater sample size will help to expand our knowledge about the role of higher order cognitive mechanisms, such as attentional processes, in motor functioning and to better understand the link between attentional dysfunction and NSS in individuals with HFA.

## Summary

Attention deficits and motor impairment have been widely reported in patients with ASD, including individuals with HFA. In this study we evaluated the functioning of multiple components of attention (i.e., alertness, selective, divided and sustained attention) and motor sign in a sample of children with HFA compared to healthy children. Furthermore, we explored the putative relationship between attentional and motor performance in HFA. The results of our study revealed that patients with HFA exhibit impairments in several attention domains as well as motor performance in comparison with typically developing children. In addition, we found strong correlation between inattentional phenomena and neurological soft sign, suggesting that altered attentional processing may be related to abnormalities of salient components of motor function in HFA.

## Electronic supplementary material

Below is the link to the electronic supplementary material.

Supplementary material 1 (DOCX 13 kb)
