# Supplementary material for: Attention Deficits Influence the Development of Motor Abnormalities in High Functioning Autism
Source: Child Psychiatry Hum Dev. 2020 Nov 4;52(6):1131–42. doi: 10.1007/s10578-020-01088-0 (PMC8528792; doi:10.1007/s10578-020-01088-0)
Supplement: Supplementary file 1 — Supplementary material 1 (DOCX 13 kb) [file 10578_2020_1088_MOESM1_ESM.docx]

**Supplementary Material**

**Methods**

***Non parametric statistical analysis***

In order to account for the small size of our samples (n=15) and confirm our results, we also performed non parametric statistic tests. The Mann-Whitney U-test (Z-test) was used to compare differences between the clinical and control groups in regard to the continuous variables collected for each subject enrolled for this study. Moreover, thee Kendall’s rank correlation coefficient or Kendall’s τ was used to find a correlation among the multiple independent variables examined.

**Results**

***Demographical variables***

In our study patients and healthy controls did not differ in terms of age (Z=-1.252; p=0.210) and IQ (Z=-0.666; p=0.505).

***Neurological soft signs***

We found significant differences between the HFA and control groups with regard to total overflow movements (Z=-2.527; p=0.01), total dysrhythmia (Z=-4.709; p=0.01) and total speed of timed activities (Z=-3.402; p=0.01).

***Attentional functioning***

*Alertness*

Comparison between the HFA and control groups using Mann-Whitney-U Test revealed no significant differences with regard to reaction time (Z=-1.431; p=0.15) and number of omission errors (Z=-1.056; p=0.29) in the tonic alertness task; however, we found a significant difference between the HFA and control groups with regard to variability of reaction time (Z=-2.344; p=0.02). Moreover, the HFA group did not differ from the control group in reaction time (Z=-0.789; p=0.43) and number of omission errors (Z=0.000; p=1.00) in the phasic alertness task. A marginally significant difference was found between groups in variability of reaction time (Z=-1.909; p=0.05) of the phasic alertness task. This latter result was not revealed by the main parametric analysis described in the manuscript.

*Selective attention*

There were no significant differences between the HFA and control groups with regard to reaction time (Z=-0.415; p=0.68) and the number of omission errors (Z=-1.856; p=0.06) in the incompatibility task, but we found significant difference in the variability of reaction time (Z=-2.490; p=0.01) within the same task. This pattern of results was identical to that found using parametric statistic tests.

*Divided attention*

In the auditory task we found significant differences between the HFA and control groups with regard to variability of reaction time (Z=-2.427; p=0.01) and number of omission errors (Z=-3.205; p=0.01), but not with regard to reaction time (Z=-1.058; p=0.29). Moreover, in the visual task we found significant differences between groups with regard to variability of reaction time (Z=-2.675; p=0.01), and number of omission errors (Z=-2.407; p=0.01), but not with regard to reaction time (Z=-1.348; p=0.18). Finally, unlike main parametric analysis, patients with HFA did not differ from healthy children in the total number of commission errors (Z=-1.383; p=0.17).

*Sustained attention*

In the sustained attention task the HFA group did not differ from the control group in reaction time (Z=-0.062; p=0.95) and variability of reaction time (Z=-1.825; p=0.07), but the patients with HFA showed a greater number of both omission (Z=-3.889; p=0.01) and commission errors (Z=-4.466; p=0.01) when compared to the healthy controls. This pattern of results was identical to that found using parametric statistic tests.
